# KATANIN-mediated microtubule severing is required for MTOC organisation and function in *Marchantia polymorpha*

**DOI:** 10.1242/dev.202672

**Published:** 2024-05-01

**Authors:** Sarah T. Attrill, Liam Dolan

**Affiliations:** ^1^ Gregor Mendel Institute, Dr Bohr-Gasse 3, Vienna 1030, Austria; ^2^ Department of Biology, University of Oxford, South Parks Road, Oxford OX1 3RB, UK

**Keywords:** Katanin, Marchantia, Microtubule, Microtubule organising centres, Polar organisers

## Abstract

Microtubule organising centres (MTOCs) are sites of localised microtubule nucleation in eukaryotic cells. Regulation of microtubule dynamics often involves KATANIN (KTN): a microtubule severing enzyme that cuts microtubules to generate new negative ends, leading to catastrophic depolymerisation. In *Arabidopsis thaliana*, KTN is required for the organisation of microtubules in the cell cortex, preprophase band, mitotic spindle and phragmoplast. However, as angiosperms lack MTOCs, the role of KTN in MTOC formation has yet to be studied in plants. Two unique MTOCs – the polar organisers – form on opposing sides of the preprophase nucleus in liverworts. Here, we show that KTN-mediated microtubule depolymerisation regulates the number and organisation of polar organisers formed in *Marchantia polymorpha*. Mp*ktn* mutants that lacked KTN function had supernumerary disorganised polar organisers compared with wild type. This was in addition to defects in the microtubule organisation in the cell cortex, preprophase band, mitotic spindle and phragmoplast. These data are consistent with the hypothesis that KTN-mediated microtubule dynamics are required for the *de novo* formation of MTOCs, a previously unreported function in plants.

## Teaser

**Summary:** Microtubule severing by KATANIN controls the number, arrangement and function of microtubule organising centres – including liverwort-specific polar organisers – in *Marchantia* cells.

## INTRODUCTION

Microtubule organising centres (MTOCs) are sites of microtubule nucleation in eukaryotic cells. The centrosomes in animal cells contain centrioles that nucleate microtubules to form the mitotic spindle and astral arrays (Bettencourt-Dias and Glover, 2007). Centrioles also form in the motile spermatozoids of sperm-producing plants – bryophytes, lycophytes and monilophytes – but are not present in the somatic cells of any land plant. However, acentrosomal MTOCs form in the somatic cells of bryophytes (Brown and Lemmon, 2011; Buschmann and Zachgo, 2016). In the liverwort, *Marchantia polymorpha*, two MTOCs – the polar organisers – form at opposite sides of the preprophase nucleus (Brown and Lemmon, 1990). Polar organisers nucleate astral arrays that polymerise towards the cell cortex, and perinuclear arrays that polymerise around the nucleus. In contrast to centrioles, polar organisers are not inherited but form *de novo* in each cell by aggregation of smaller microtubule nucleation sites (foci) during preprophase (Buschmann et al., 2016). At the onset of mitosis, polar organisers disassemble and their γ-tubulin relocates to the poles of the mitotic spindle (Brown and Lemmon, 2011; Brown et al., 2004). Nothing is known about the proteins required for the formation, organisation or function of polar organisers.

KATANIN (KTN) is a microtubule-severing enzyme that remodels microtubule networks and alters microtubule dynamics. KTN is a highly conserved AAA ATPase composed of a regulatory p80 subunit and a catalytic p60 subunit (McNally and Vale, 1993). Using free energy from ATP hydrolysis, KTN severs microtubules to generate free negative ends that undergo catastrophic depolymerisation (McNally and Roll–Mecak, 2018). In animals, KTN regulates mitotic spindle length and centriole number (Hu et al., 2014; McNally et al., 2006). In the angiosperm *Arabidopsis thaliana*, KTN preferentially cuts microtubules in the cell cortex at points of branching and crossover, destabilising these discordant microtubules and generating parallel cortical arrays (Deinum et al., 2017; Zhang et al., 2013). This, in turn, orients cellulose deposition and restricts plant cell growth to a single direction (Burk and Ye, 2002). KTN further regulates preprophase band (PPB) formation, mitotic spindle mobility and phragmoplast expansion in *A. thaliana* to orient the cell division plane (Komis et al., 2017; Panteris et al., 2011). Although KTN-mediated severing is required to generate dynamic cortical and mitotic microtubule arrays in plants, it reveals nothing about the function of KTN in the formation of plant MTOCs. This is because, unlike bryophytes, stable MTOCs do not form in angiosperm cells (Chan et al., 2003).

We set out to test the hypothesis that KTN-mediated microtubule severing is required for the formation of polar organisers in *M. polymorpha*. First, we show that KTN organises microtubule arrays during interphase, mitosis and cytokinesis, indicating multiple conserved functions between *M. polymorpha* and *A. thaliana*. We also demonstrate that KTN regulates the number and organisation of polar organisers in *M. polymorpha,* supporting a previously unreported function for KTN in plants during the *de novo* formation of MTOCs.

## RESULTS AND DISCUSSION

### There is a single *KTN* gene in *Marchantia polymorpha* and mutations in Mp*KTN* result in defective organ development

To define the function of KTN in polar organiser formation, we characterised the phenotype of mutants carrying loss-of-function mutations in the Mp*KTN* gene. To identify *KTN* genes in *Marchantia polymorpha*, the protein sequence for the *A. thaliana KTN* p60 subunit*,* AT1G80350, was used as a query in a BLASTp search against the *M. polymorpha* proteome. Mapoly0116s0028 (Mp4g20260) was the most similar sequence identified. Two phylogenetic trees were then constructed using KTN and KTN-LIKE protein sequences from a variety of land plant and algal species (Fig. S1A,B). These sequences were identified using the *M. polymorpha* sequence as a query in BLASTp searches across multiple databases (Table 1). The topology of the two trees indicates that Mapoly0116s0028 (Mp4g20260) is a KTN family member and is the only *KTN* p60 subunit gene in *M. polymorpha* (Fig. S1A,B). This gene will now be referred to as Mp*KTN*.

**Table 1.**
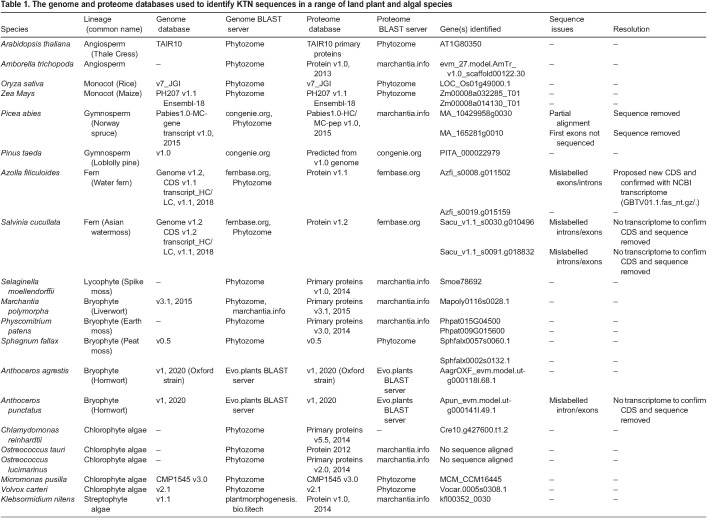
The genome and proteome databases used to identify KTN sequences in a range of land plant and algal species

**Fig. 1.**
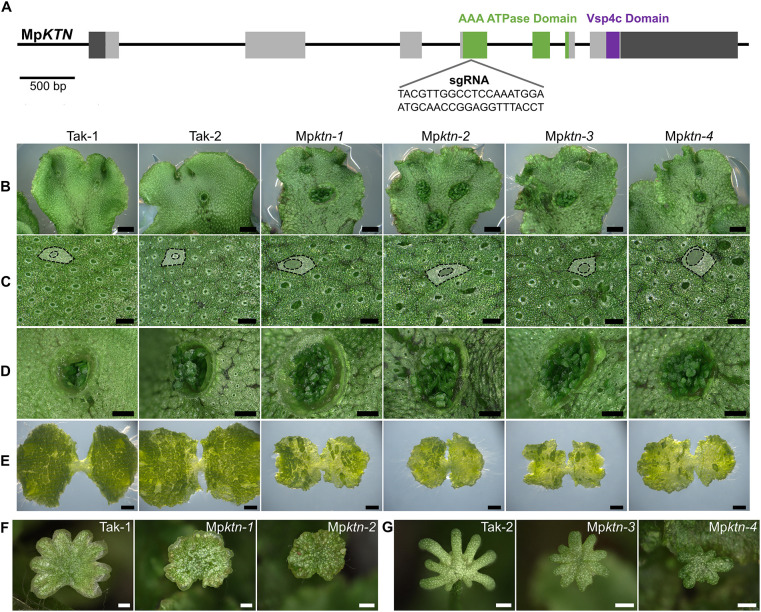
**KTN is required for tissue and organ development in *M. polymorpha*.** (A) Schematic of the Mp*KTN* gene indicating one sgRNA location and sequence. Light-grey boxes indicate exons; dark-grey boxes indicate untranslated regions. Green indicates regions encoding the AAA ATPase domain. Purple indicates the region encoding the Vsp4c domain. Scale bar: 500 bp. (B) Dorsal thallus of Tak-1, Tak-2 and four Mp*ktn* mutants. Scale bars: 2 mm. (C) Dorsal epidermal surface of Tak-1, Tak-2 and four Mp*ktn* mutants. An air chamber and air pore are outlined and false coloured in each image. Scale bars: 300 μm. (D) Gemma cups on the dorsal thallus of Tak-1, Tak-2 and four Mp*ktn* mutants. Scale bars: 1 mm. (E) 9-day-old gemmalings from Tak-1, Tak-2 and four Mp*ktn* mutants. Scale bars: 1 mm. (F) Antheridiophores of Tak-1 and two male Mp*ktn* mutants grown under far-red light conditions. Scale bars: 1 mm. (G) Archegoniophores of Tak-2 and two female Mp*ktn* mutants grown under far-red light conditions. Scale bars: 1 mm.

To investigate the function of KTN in polar organiser formation, we used CRISPR/Cas9 mutagenesis to generate loss-of-function mutations in Mp*KTN*. sgRNAs were designed to target regions in the Mp*KTN* gene encoding the AAA ATPase catalytic domain and Vsp4C domain (Fig. 1A; Fig. S1C). Mutations in these regions of the *KTN* gene in *A. thaliana* result in a loss of KTN function (Luptovčiak et al., 2017). A series of Mp*KTN* mutant alleles – including nucleotide deletions, insertions and substitutions – were produced that altered the predicted amino acid sequence by inducing frameshifts, substitutions and protein truncations (Fig. S1D,E). All Mp*ktn* mutants developed similar phenotypes with multiple developmental defects compared with wild type. This included a crinkled thallus, enlarged air chamber pores, enlarged gemma cups, irregular gemmaling development, delayed plant growth and abnormal reproductive organs (Fig. 1B-G; Fig. S2; Fig. S3). Many of the developmental defects reflect those in At*ktn* (Luptovčiak et al., 2017). The consistent defective phenotype of all Mp*ktn*, in combination with the mutations being located within regions encoding highly conserved functional domains, suggests that Mp*ktn* have a complete loss of KTN function.

### KTN activity is required for the organisation of cortical arrays in the gemma epidermis

**Fig. 2.**
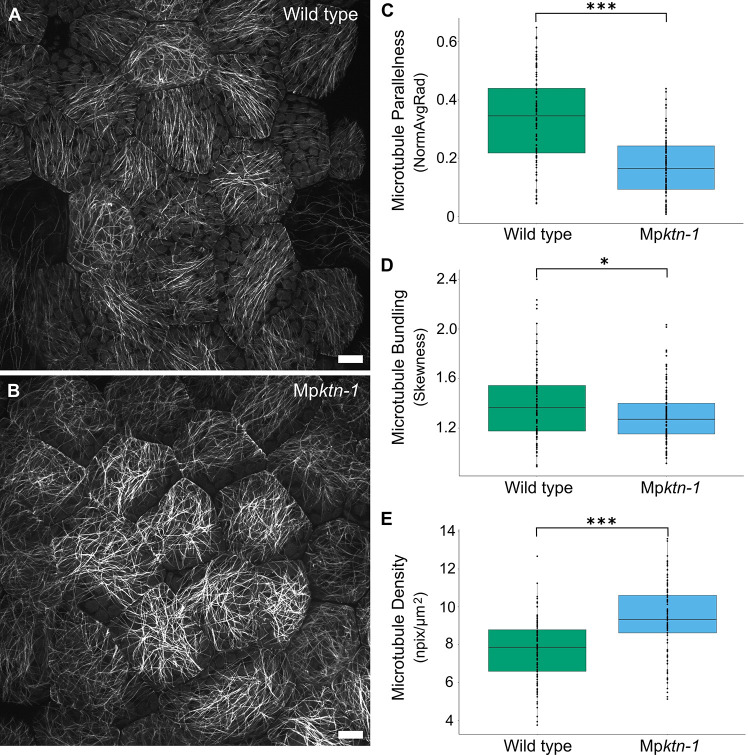
**KTN promotes parallelness and bundling of cortical microtubules.** (A,B) Cortical microtubule arrays in central epidermal cells of wild-type (A) and Mp*ktn-1* (B) 2-day-old gemmalings. Images are *z*-projections. Scale bars: 10 μm. (C-E) Boxplots of the parallelness (C), bundling (D) and density (E) of cortical microtubules in wild-type and Mp*ktn-1* epidermal cells. Wild type, *n*=108 cells; Mp*ktn-1, n*=107 cells. Cells from 10 gemmalings per genotype were examined. Each parameter was analysed using Welch's two-tailed *t*-tests to compare differences between wild type and Mp*ktn-1* (*P≤0.05 and ***P≤0.001).

KTN-mediated microtubule severing is required for the organisation of microtubules in the cortex of *A. thaliana* cells (Komis et al., 2017). To test whether KTN controls microtubule organisation in *M. polymorpha*, the arrangement of cortical arrays in Mp*ktn* was investigated. Mp*ktn-1* and Mp*ktn-2*, which harbour mutations in the Mp*KTN* gene at the AAA ATPase-encoding domain, were crossed with wild-type (Mp*KTN*) plants expressing the microtubule reporter *p*Mp*EF1α:GFP-*Mp*TUB1* (Buschmann et al., 2016). Cas9-free wild-type and Mp*ktn* expressing GFP-MpTUB1 were selected from the F1 progeny (Fig. S1F). Microtubules in the cortex of the outer periclinal wall of the epidermal cells at the centre of 2-day-old gemmalings were imaged. In wild type, two arrangements of cortical arrays were observed: some cells developed parallel arrays, whereas other cells developed non-parallel arrays (Fig. 2A; Fig. S4A). By contrast, cortical arrays were consistently randomly organised in all cells of Mp*ktn-1* and Mp*ktn-2* (Fig. 2B; Fig. S4B). Quantification revealed significant differences in microtubule parallelness (the orientation of microtubules relative to one another within a cell) and in microtubule bundling (the formation of bundles from parallel microtubule arrays) between wild type and Mp*ktn-1*. Both measures were, on average, lower in Mp*ktn-1* than in wild type, but with higher variation between cells (Fig. 2C,D; Fig. S4C). Microtubule density (the area of the dorsal cell surface covered by microtubules) was consistently greater in Mp*ktn-1* cells than in wild type (Fig. 2E; Fig. S4C). Cell area was similar between Mp*ktn-1* and wild type, but cell shape was more variable in Mp*ktn-1* (Fig. S4C-E). Together, these data indicate that KTN-mediated microtubule depolymerisation is required for the organisation of cortical microtubules into parallel, bundled, low-density arrays in *M. polymorpha*. Furthermore, these data demonstrate that MpKTN generates variation in the microtubule organisation between individual epidermal cells.

### KTN is required for the formation of two compact polar organisers per cell

**Fig. 3.**
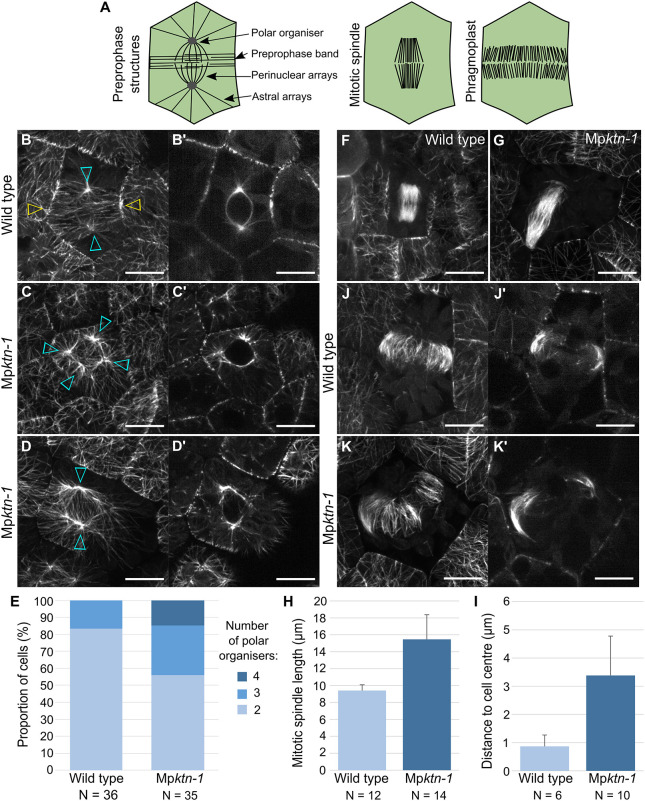
**KTN regulates the number and organisation of polar organisers in each cell.** (A) Microtubule organisations in *M. polymorpha* cells during preprophase, mitosis and cytokinesis. (B-D′) Polar organisers and astral arrays in wild-type (B,B′) and two Mp*ktn-1* (C-D′) cells. Images are *z*-projections (B-D) and central slices in *xy* (B-D′). Scale bars: 10 μm. Cyan arrowheads indicate polar organisers; yellow arrowheads indicate the preprophase band. (E) Proportion of wild-type and Mp*ktn-1* cells with two, three or four polar organisers. Wild type, *n*=36 cells; Mp*ktn-1*, *n*=35 cells. (F,G) Mitotic spindle in wild-type (F) and Mp*ktn-1* (G) cells. Images are *z*-projections. Scale bars: 10 μm. (H) Graph of the mitotic spindle length (μm) in wild type and Mp*ktn-1*. Wild type, *n*=12 spindles; Mp*ktn-1*, *n*=14 spindles. (I) Graph of the distance (μm) between the centre of the mitotic spindle and the centre of the cell for wild type and Mp*ktn-1.* Wild type, *n*=6 spindles; Mp*ktn-1*, *n*=10 spindles. (J-K′) The phragmoplast in wild-type (J,J′) and Mp*ktn-1* (K,K′) cells. Images are *z*-projections (J,K) and central slices in *xy* (J′,K′). Scale bars: 10 μm.

As MTOCs – the sites of localised microtubule nucleation – are not present in *A. thaliana*, the role of KTN-mediated microtubule severing in MTOC formation has not been established in land plants (Luptovčiak et al., 2017). However, two MTOCs, known as polar organisers, form *de novo* at opposite poles of the preprophase nucleus in *M. polymorpha* (Brown and Lemmon, 2011; Buschmann et al., 2016). Polar organisers nucleate astral microtubules that polymerise into the cell cortex, and perinuclear microtubules that polymerise towards the nucleus equator (Fig. 3A). We hypothesised that KTN-mediated microtubule severing would be required for the formation and organisation of polar organisers.

To test the hypothesis that KTN is required for polar organiser formation, the polar organiser in preprophase cells near the meristematic notch of 2-day-old gemmalings were imaged. Wild-type cells formed two distinct polar organisers at opposite sides of the nucleus (Fig. 3B; Movie 1). By contrast, Mp*ktn-1* cells often formed more than two polar organisers, resulting in a multipolar structure around the nucleus (Fig. 3C,D; Movie 2). Quantification showed that wild type had two, or occasionally three, polar organisers per cell (Fig. 3E). By contrast, Mp*ktn-1* had up to four distinct polar organisers per cell. In wild type, the astral microtubules radiated symmetrically towards the cell cortex and the perinuclear microtubules formed a bipolar array (Fig. 3B). By contrast, both astral and perinuclear arrays were denser and relatively disorganised in Mp*ktn-1* (Fig. 3C,D). Furthermore, the astral microtubules radiated asymmetrically towards the cell cortex in Mp*ktn-1*. Overall, these data indicate that KTN-modulated microtubule severing is required for the organisation of two polar organisers at opposing sides of the nucleus.

The PPB is a parallel microtubule array that forms in the cell cortex during preprophase – after polar organiser formation – and fine-tunes the positioning of the cell division plane (Brown and Lemmon, 1990; Rasmussen et al., 2013; Schaefer et al., 2017). A PPB formed in most wild-type cells with two polar organisers (Fig. 3B). In Mp*ktn-1* cells with polar organisers, no distinctive PPB formed, but microtubules were present at the cell cortex in a random organisation (Fig. 3C,D). This suggests that the initial steps of forming a precise, parallel microtubule array – such as the PPB – are KTN dependent in *M. polymorpha*.

To investigate the role of KTN in mitotic spindle and phragmoplast formation, microtubules in dividing wild-type and Mp*ktn-1* cells were imaged. Mitotic spindles were short, box-shaped and centrally positioned in wild-type cells (Fig. 3F). By contrast, the mitotic spindles were significantly longer and had tapered ends in Mp*ktn-1* (Fig. 3G,H). Similar elongated spindles were observed on inhibition or knockdown of KTN in *Caenorhabditis elegans* embryos, mouse oocytes and *Xenopus tropicalis* cells (Gao et al., 2019; Loughlin et al., 2011; McNally et al., 2006). The position of mitotic spindles was highly variable in Mp*ktn-1* and significantly further from the cell centre than in wild type (Fig. 3G,I). This spindle mispositioning in Mp*ktn-1* cannot be explained by differences in cell geometry alone. Phragmoplasts in wild type extended straight across the cell to divide the cell into two relatively equal parts (Fig. 3J). By contrast, phragmoplasts in Mp*ktn-1* were often bent along their length and positioned to divide the cell into two unequal parts (Fig. 3K). We conclude that KTN regulates mitotic spindle length and position, as well as phragmoplast morphology and position in *M. polymorpha.*

Overall, we conclude that KTN is required for the organisation of polar organisers and microtubule arrays in the PPB, mitotic spindle and phragmoplast in *M. polymorpha*. Polar organisers form by aggregation of smaller foci around the nucleus into two bipolar centres, as described by Buschmann et al. (2016). Our data are consistent with the hypothesis that KTN-mediated severing, and the resulting catastrophic depolymerisation of microtubules, is required for the aggregation of multiple foci into two polar organisers. We further hypothesise that KTN-mediated severing is similarly required for the reorganisation of cortical microtubules into a PPB.

### KTN controls the position of the new cell plate by aligning the mitotic spindle axis with the polar organiser axis

It has been proposed that, together, the PPB and polar organisers determine the position of the mitotic spindle and phragmoplast, and ultimately the plane of cell division in *M. polymorpha* (Buschmann et al., 2016). We hypothesised that in Mp*ktn*, the disordered polar organisers were the origin of the mispositioned mitotic spindles and phragmoplasts. This would result in abnormal cell division planes in Mp*ktn*. To investigate whether polar organisers orient the cell division plane, timelapse imaging of microtubule organisation in dividing wild-type and Mp*ktn-1* cells was performed.

**Fig. 4.**
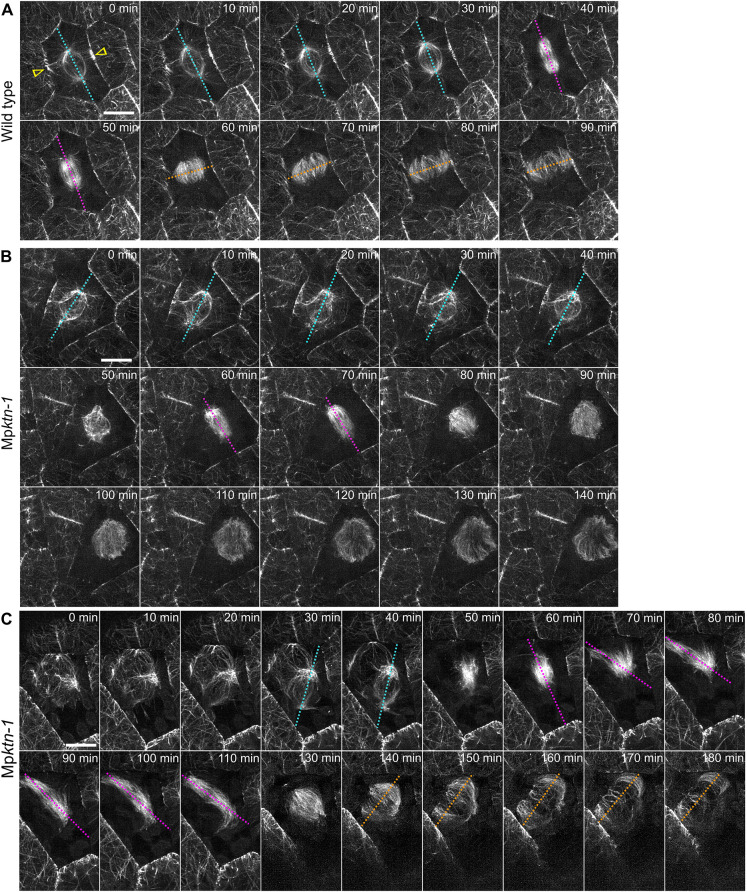
**The polar organiser axis and mitotic spindle axis misalign in dividing Mp*ktn-1* cells.** (A-C) Timelapses of microtubules in dividing wild-type (A) and Mp*ktn-1* (B,C) cells from 2-day-old gemmalings. Images are deconvolved *z*-projections taken at 10-min intervals. Dotted cyan lines indicate the polar organiser axis; dotted magenta lines indicate the mitotic spindle axis; dotted orange lines indicate the phragmoplast axis. Yellow arrowheads indicate the preprophase band. Scale bars: 10 μm.

We compared the relationship between the orientation of the polar organiser axis (the axis between the two polar organisers), the mitotic spindle axis (the axis between the two spindle poles) and the plane of phragmoplast expansion in each cell. In wild type, the polar organisers and their associated perinuclear arrays were positioned near the cell centre (0 to 30 min in Fig. 4A). The polar organiser axis was parallel to the dorsal cell surface and perpendicular to the PPB. Later, the mitotic spindle formed in the cell centre with an axis parallel to the polar organiser axis and remained in this orientation throughout mitosis (40 to 50 min in Fig. 4A). These observations of polar organiser dynamics during wild-type cell division were identical to those previously described by Buschmann et al. (2016). In Mp*ktn-1*, it was difficult to define the polar organiser axis as there was often supernumerary polar organisers and a disorganised, multipolar perinuclear array. We therefore defined the polar organiser axis as a line between the two brightest microtubule foci. In Mp*ktn-1*, the polar organiser axis was often tilted within the cell, i.e. not parallel to the dorsal cell surface (0 to 40 min in Fig. 4B,C). When the mitotic spindle formed, its axis was oblique relative to the polar organiser axis (clearly viewed in the *xz*-plane at 50 to 80 min in Fig. S5D). Furthermore, the mitotic spindle elongated and rotated overtime in Mp*ktn-1* (60 to 110 min in Fig. 4C). This indicates that the spatial relationship between the polar organiser axis and the mitotic spindle axis are uncoupled in Mp*ktn-1*.

The phragmoplast formed and expanded in a plane perpendicular to the last position of the mitotic spindle in both wild-type and Mp*ktn-1* cells. In wild type, the phragmoplast formed in the cell centre and expanded in the same plane as the PPB (60 to 90 min in Fig. 4A). The new cell wall formed perpendicular to the dorsal surface of the cell and split the cell into two more or less equal halves, i.e. an anticlinal symmetric division (90 min in Fig. S5A). By contrast, in Mp*ktn-1*, as the mitotic spindle was not oriented parallel to the dorsal surface of the cell and/or rotated before cytokinesis, the phragmoplast divided the cell into two unequal parts (140 to 180 min in Fig. 4C) or oblique relative to the dorsal surface of the cell (clearly viewed in the *yz*-plane at 90 to 140 min in Fig. S5B). These data indicate that KTN-mediated microtubule severing is required to orient the mitotic spindle and the subsequent phragmoplast for symmetrical anticlinal divisions.

Taken together, our data demonstrate that KTN-mediated microtubule severing is required for the bipolar organisation of MTOCs in *M. polymorpha*. This is a previously unreported function for KTN in land plants. We also show that severing of microtubules by KTN, leading to microtubule depolymerisation, is required for organisation of cortical arrays. We further show that KTN is required to orient cell divisions through its functions in PPB formation, MTOC positioning, and mitotic spindle alignment and stabilisation. Roles for KTN in the dynamic reorganisation of cortical and mitotic microtubule arrays have previously been described in the angiosperm *A. thaliana* (Lindeboom et al., 2013; Komis et al., 2017). However, the role of KTN in plant MTOC organisation and function has never been described.

Bryophytes develop MTOCs – localised sites of microtubule nucleation – unlike other land plants that develop delocalised sites of microtubule polymerisation (Chan et al., 2003; Brown and Lemmon, 2011). Polar organisers are liverwort-specific MTOCs that contain γ-tubulin and nucleate two distinct populations of microtubules: the astral arrays and the perinuclear arrays (Brown et al., 2004; Buschmann et al., 2016). In loss-of-function Mp*ktn* mutants, polar organisers were generally larger, more elongated and nucleated denser arrays than in wild-type cells. We therefore speculate that KTN-mediated microtubule depolymerisation is required to restrict the size of each polar organiser (Fig. S6A). This is consistent with the hypothesis that the polar organiser size is proportional to the number of attached microtubules.

We also speculate that KTN-mediated microtubule depolymerisation is required for the development of bipolar pairs of polar organisers. Two polar organisers form in each wild-type cell through the fusion of microtubule foci in a 3 h period before mitosis, a process termed bipolar aggregation by Buschmann et al. (2016). We show that supernumerary polar organisers form in Mp*ktn* cells. This indicates that without functional KTN, the number of microtubule foci often did not reduce to two before mitosis. If the bipolar aggregation model proposed by Buschmann et al. is correct, our data would be consistent with the hypothesis that KTN-mediated microtubule depolymerisation is required for the aggregation and/or fusion of microtubule foci into polar organisers (Fig. S6B).

Together, these data demonstrate that KTN-mediated microtubule dynamics are required for the bipolar organisation of MTOCs in *M. polymorpha*. We predict that this is achieved by regulating the *de novo* formation of MTOCs. This represents a previously unreported function for KTN in land plants.

## MATERIALS AND METHODS

### Sequence alignments and generation of phylogenetic trees

The protein sequence for the *A. thaliana KTN* p60 subunit, AT1G80350, was used in a BLASTp search against the *M. polymorpha* proteome. The top hit, Mapoly0116s0028 (Mp4g20260), was used as the query sequence for BLASTp searches in the proteome databases of 20 land plant and algal species (Table 1). In each species, except two *Osterococcus* species, sequences with E-values above E-87 were identified and selected. The protein sequences were aligned using MAFFT version 7 employing the L-INS-i method (Katoh et al., 2002). Sequences from three species – *Anthoceros punctatus, Salvinia cucullata* and *Picea abies* – were subsequently removed due to suspected mis-annotation of one or more exon-intron boundaries, or incomplete sequencing. For one species – *Azolla filiculoides* – a new coding sequence was proposed after suspected mis-annotation of the exon-intron boundaries, confirmed by the transcriptome. Sequences from 15 species were realigned in MAFFT and trimmed using BioEdit software to regions encoding the conserved AAA ATPase and Vsp4C domains. These domains were identified using the SMART protein domain dataset (Letunic and Bork, 2018). Maximum likelihood trees were generated using MEGA-X 10 software using all amino acid sites, and bootstrap values were calculated from 500 replicates (Kumar et al., 2018).

### Plant lines, growth conditions and crossings

The wild-type *Marchantia polymorpha* accessions used were Takaragaike-1 (Tak-1) and Takaragaike-2 (Tak-2). All constructs were transformed into the progeny derived from crossing Tak-1 and Tak-2. Plants were grown on ½-strength B5 Gamborg's medium containing 1.5 g/L B5 Gamborg, 0.5 g/L MES hydrate and 1% sucrose, with pH adjusted to 5.5 and set with 1% agar. Plants were grown at 23°C in continuous white light at 50-60 μmol m²s¹.

To induce reproductive development, mature plants were potted on soil, containing a 1:3 ratio of fine vermiculite and Neuhaus N3 compost, at 20°C in long day conditions of 16 h light/8 h dark. White light was set at 50-60 μmol m²s¹ and enhanced with far-red light at 30-40 μmol m²s¹. Male and female plants were crossed to generate sporangia. Sporangia were sterilised in 1% sodium dichloroisocyanurate (NaDCC) for 3 min before washing with water and releasing the spores.

### sgRNA design for CRISPR/Cas9 mutagenesis

sgRNAs, consisting of 20 nucleotides followed by a NGG sequence, were designed to target the Mp*KTN* (Mapoly0116s0028/Mp4g20260) gene using the CRISPR-P software (Lei et al., 2014). Candidate sequences were checked for off-target hits by a BLAST search against the *M. polymorpha* genome v4 (marchantia.info). The two final sequences were sgRNA-K3 TACGTTGGCCTCCAAATGGA(GGG) – to which a CTCG overhang was added to the 5′ end before cloning – and sgRNA-K5 GGAGCTTGCCAGACGTACAG(AGG).

### Cloning of CRISPR/Cas9 plasmids

Cloning of the sgRNA-K3 Cas9 plasmid used the vectors and protocol presented by Thamm et al. (2020) (adapted from Sugano et al., 2014). Cloning of the sgRNA-K5 Cas9 plasmid was performed using vectors from the OpenPlant toolkit and following the protocol published by Sauret-Güeto et al. (2020).

### Transformation of constructs into *M. polymorpha*

Plasmids were transformed into *M. polymorpha* sporelings using transgenic *Agrobacterium* following the method developed in Ishizaki et al. (2008) and improved upon by Honkanen et al. (2016). Transgenic plants were selected by their resistance to 10 µg/ml hygromycin.

### DNA extraction and sequencing of *M. polymorpha*

DNA extraction and amplification used the Phire Plant Direct PCR kit (ThermoFisher Scientific) (PCR primers, Table 2) and GeneJet Gel Purification kit (ThermoFisher Scientific). Alternatively, DNA was extracted from 3×3 mm pieces of plant tissue ground within 100 μl of extraction buffer [100 mM Tris HCl (pH 9.5), 1 M KCl and 10 mM EDTA]. Samples were incubated at 65°C for 10 min before dilution in 500 µl MonoQ water. DNA was PCR amplified using 2× HS Taq Polymerase, 2× Hot Start MM, 0.5 µM forward PCR primer, 0.5 µM reverse PCR primer, 1 µl DNA and 7 µl nuclease-free water. The PCR products were purified using an the ExoSAP mix (0.04 µl exonuclease I, 0.4 µl shrimp alkaline phosphatase and 1.56 µl storage solution). The reaction was incubated at 37°C for 30 min, before deactivation at 80°C for 10 min. Isolated DNA was Sanger sequenced (primers in Table 2). Sequences were aligned against the Tak-1 genome sequence using Geneious or CLC Genomics Workbench to identify mutations at the protospacer adjacent motif (PAM) site.

**Table 2.**
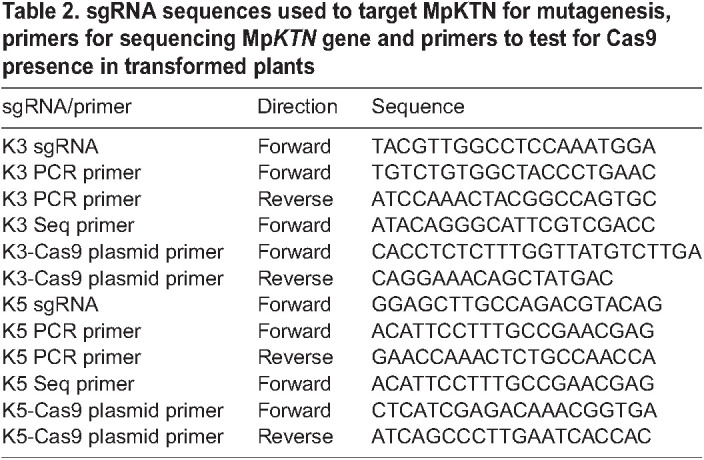
sgRNA sequences used to target MpKTN for mutagenesis, primers for sequencing Mp*KTN* gene and primers to test for Cas9 presence in transformed plants

### Generation and selection of CRISPR/Cas9 mutants expressing reporters

Mp*katanin* (Mp*ktn*) mutants were crossed to wild-type plants expressing the microtubule reporter *p*Mp*EF1α:GFP-*Mp*TUB1* (GFP-MpTUB1), generated by Buschmann et al. (2016). The resulting F1 progeny were grown, genotyped for mutations (PCR primers in Table 2) and tested for the presence of the Cas9 plasmid (Cas9 primers in Table 2). Cas9-free lines were screened for GFP-MpTUB1 by testing for resistance to 10 µg/ml hygromycin and fluorescence imaging. Cas9-free wild-type and Mp*ktn* siblings expressing the GFP-MpTUB1 reporter were selected (Fig. S1F).

### Stereomicroscope imaging of plant tissues

Gemmalings were imaged with the Leica MZ16FA stereomicroscope equipped with the Leica DFC300 FX camera. Mature plants and tissues were imaged with the Keyence VHX-7000 digital equipped with a VHX-7020 camera and VH-Z00R/T and VH-ZST lenses.

### Spinning disk imaging of microtubules

Imaging chambers were adapted from Kirchhelle and Moore (2017). A breathable gum boarder (Carolina Observation gel) was filled with Gamborg media and layered with cellophane soaked in liquid Gamborg media (½-strength B5 Gamborg's medium without agar). Perfluorodecalin and 0-day gemmae were added, and the chamber sealed with a cover slip. Gemmae were grown for 2 days within the chamber before imaging.

Microtubules were imaged with an Olympus IX3 Series (IX83) inverted microscope equipped with a Yokogawa W1 spinning disk, Hamamatsu ORCA-Fusion CMOS camera and a 100×/1.45 NA oil objective. Samples were excited at 488 nm and emission captured at 525 nm. *Z*-stacks were taken with 0.26 µm slices. GFP-MpTUB1 labelled cortical arrays were imaged in the central epidermal cells, and mitotic arrays in dividing cells near the meristem.

### Deconvolution and conversion of spinning disk images

Image deconvolution used Huygens software (Scientific Volume Imaging). *Z*-projections and central slices were converted using ImageJ Fiji (Schindelin et al., 2012).

### Analysis of microtubule organisation

Cortical microtubules were analysed using the ImageJ LPX package published by Higaki et al. (2010), following the steps in Higaki (2017). From *z*-projections of the central epidermis, individual cells were outlined using the Freehand Tool in ImageJ Fiji. Microtubules were skeletonised using the LPX Filter2d with the Otsu method and a line extract value of 5. The image was masked by the cell outlines to identify the skeletonised microtubules in each cell for analysis using the LPX script. This measured the microtubule parallelness, bundling and density per cell, and the shape and circularity of each cell. Parallelness was measured as the distribution of angles formed by microtubules (NormAvgRad) ranging from 0 (non-parallel) to 1 (parallel). Bundling was measured by the skewness in the fluorescence intensity distribution along a microtubule, with higher intensity values indicating higher bundling. Density was measured as the number of segmented pixels in a cell divided by the cell area (npix/µm²). Welches two-tailed *t*-tests and two-tailed *F*-tests were performed in Microsoft Excel to identify significant differences in the mean and variability between wild type and mutant for each parameter measured. Boxplots were produced in R.

The number of polar organisers per cell were manually counted from *z*-projections. Spindle length was measured in ImageJ Fiji using the Line tool from *z*-projections. Spindle position was quantified by identifying the central point of the cell and spindle in ImageJ Fiji, then calculating the distance between these points. Statistical analysis was carried out using Microsoft Excel.

### Quantification of plant tissue area

Gemmalings were imaged every 2 days with the Berthold NightOwl II LB 983 *In Vivo* Imaging system, which captures chlorophyll autofluorescence of living tissue after exposure to 120 s white light. Tissue area was detected and quantified using the indiGo software package. A growth curve was generated using ggplot2 and drc packages in R (Wickham, 2009).

## Supplementary Material



10.1242/develop.202672_sup1 Supplementary information
