# Supplementary material for: KATANIN-mediated microtubule severing is required for MTOC organisation and function in Marchantia polymorpha
Source: Development. 2024 May 1;151(20):dev202672. doi: 10.1242/dev.202672 (PMC11112166; doi:10.1242/dev.202672)
Supplement: Supplementary information [file develop-151-202672-s1.pdf]

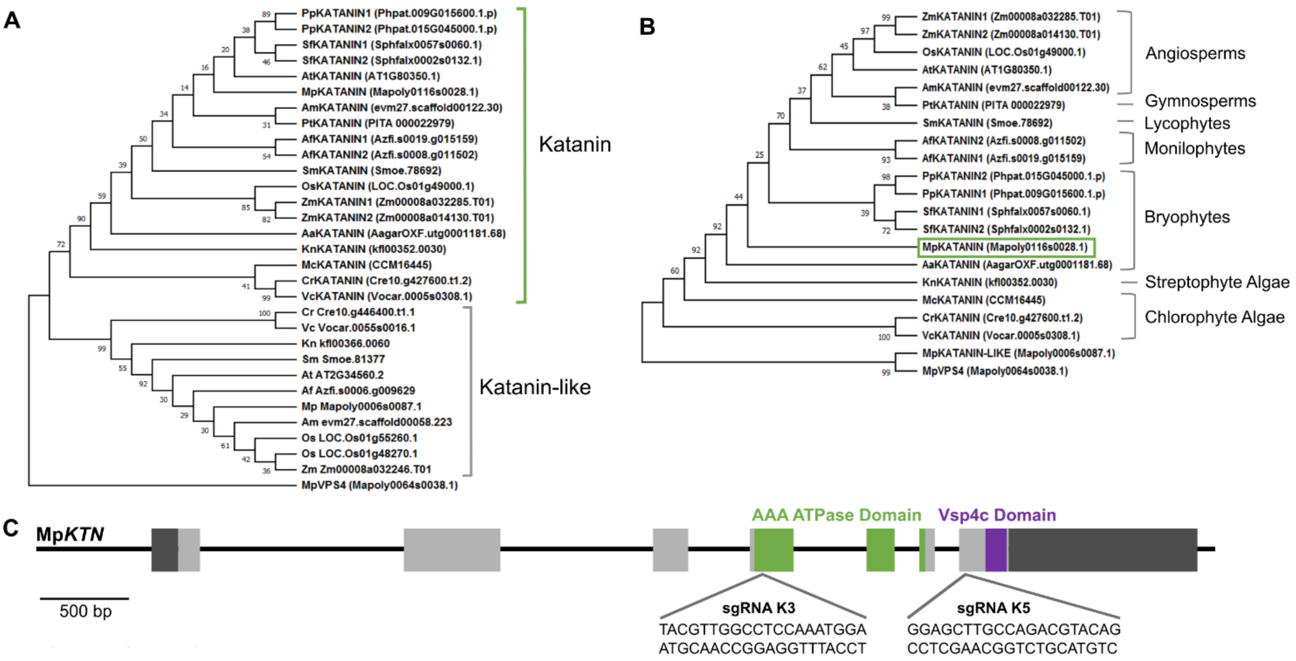

D

Plant line	sgRNA	Gene sequence	Genetic consequence	Predicted protein sequence	Predicted protein consequence
Wild type		ACGTTGGCCTCCAAATGGAGGGGTGAG		TLASKWRGE	
ktn-1	K3	ACGTTGGCCTCCAACGAACGGAGGGGT	3 bp insertion, T>C substitution	TLASNERRGE	Lys >Asn & Trp>Arg substitutions, additional Glu
ktn-2	K3	ACGTTGGCCTCCAAATTGGAGGGGTGA	1 bp insertion	TLASKLEG*	Frameshift, truncation 4 AA downstream
ktn-3	K3	ACGTTGGCCTCCAAATGGGAGGGGTG	1 bp insertion	TLASKWEG*	Frameshift, truncation 3 AA downstream
ktn-4	K3	ACGTTGGCCTCCAAACGTAGGAGGGGT	3 bp insertion, T>A substitution	TLASKRRGE	Trp>Arg substitution, additional Arg
ktn-5	K3	ACGTTGGCCTCCAA--------GAGA	8 bp insertion, T>A substitution	TLASKRE...*	Frameshift, truncation 23 AA downstream
ktn-6	K3	TCCAAACAAACAAACCCAAGCCTCTCATGG	21 bp insertion	TLASKQTNPSLSWRGE	Insertion of QTNPSLS, no frameshift
ktn-7	K3	TCCAAACGTAGCTGGACGTAGGTTGGA	17 bp insertion	TLASKRSWT*	Insertion of RSWT and STOP codon, truncation
ktn-8	K3	ACGTTGGCCTCGTA--GGAGGGGTGA	CA>GT substitution, 2 bp deletion	TLAS*	STOP codon formed, immediate truncation
Wild type		GAGGAGCTTGCCAGACGTACAGAGGGT		EELARRETG	
K5-15	K5	GAGCTTGCCAGACGTATAGCTTGCCAG	8 bp insertion	EELARRIA...*	Frameshift, truncation 11 AA downstream
K5-45	K5	GAGGAGCCCTTGCCA-------------	169 bp deletion	EEPLPKFHA...	Large deletion, frameshift
K5-58	K5	GAGGAGCTTGCCAGA--------GGG	8 bp deletion	EELARGL*	Frameshift, truncation 2 AA downstream

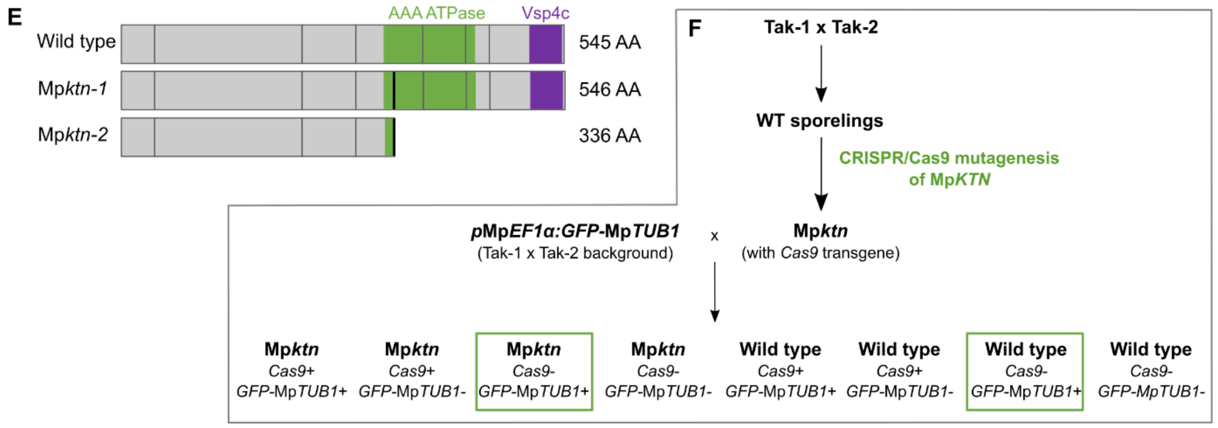

Fig. S1. *Marchantia polymorpha* has a single *KTN* gene. (A) Maximum likelihood tree of the KTN and KTN-LIKE proteins from 15 land plant and algal species, based on the aligned AAA domain of protein sequences. Includes MpVSP4 (Mapoly0064s0038) protein as an outgroup. The KTN and KTN-like clades are labelled. (B) Maximum likelihood tree of KTN protein from 15 plant and algal species, based on the aligned AAA and Vsp4c domains of protein sequences. Includes MpVSP4 (Mapoly0064s0038) and MpKTN-LIKE

Development • Supplementary information

(Mapoly0006s0087) proteins. The major plant lineages are labelled and the single Mp*KTN* (Mapoly0116s0028) is indicated by a green box. At, *Arabidopsis thaliana.* Am, *Amborella trichopoda.* Os, *Oryza sativa.* Zm, *Zea mays*. Pt, *Pinus taeda.* Af, *Azolla filiculoides.* Sm, *Selaginella moellendorffi.* Mp, *Marchantia polymorpha.* Pp, *Physcomitrium patens.* Sf, *Sphagnum fallax.* Aa, *Anthoceros agrestis*. Cr, *Chlamydomonas reinhardtii.* Mc, *Micromonas pusilla.* Vc, *Volvox carteri*. Kn, *Klebsormidium nitens.* (C) Schematic of the Mp*KTN* gene indicating the location and sequences of the two sgRNAs (K3 and K5). Light grey boxes indicate exons. Dark grey boxes indicate untranslated regions. Green indicates regions encoding the AAA ATPase domain. Purple indicates the region encoding the Vsp4c domain. Scale bar: 500 bp. (D) Table of Mp*ktn* mutants including their mutated gene sequences and predicted protein sequences. Red indicates a difference in nucleotide or amino acid sequence compared to the wild type (Tak-1) sequence. (E) Diagram of the MpKTN protein structure and length in wild type and three selected Mp*ktn* mutants. Grey boxes indicate exons. Green regions indicate the AAA ATPase domain. Purple regions indicate the Vsp4c domain. Black regions indicate mutated sequences. (F) Diagram showing the generation of Cas9-free Mp*ktn* mutants expressing the microtubule reporter, *p*Mp*EF1α:GFP*-Mp*TUB1* (GFP-MpTUB1).

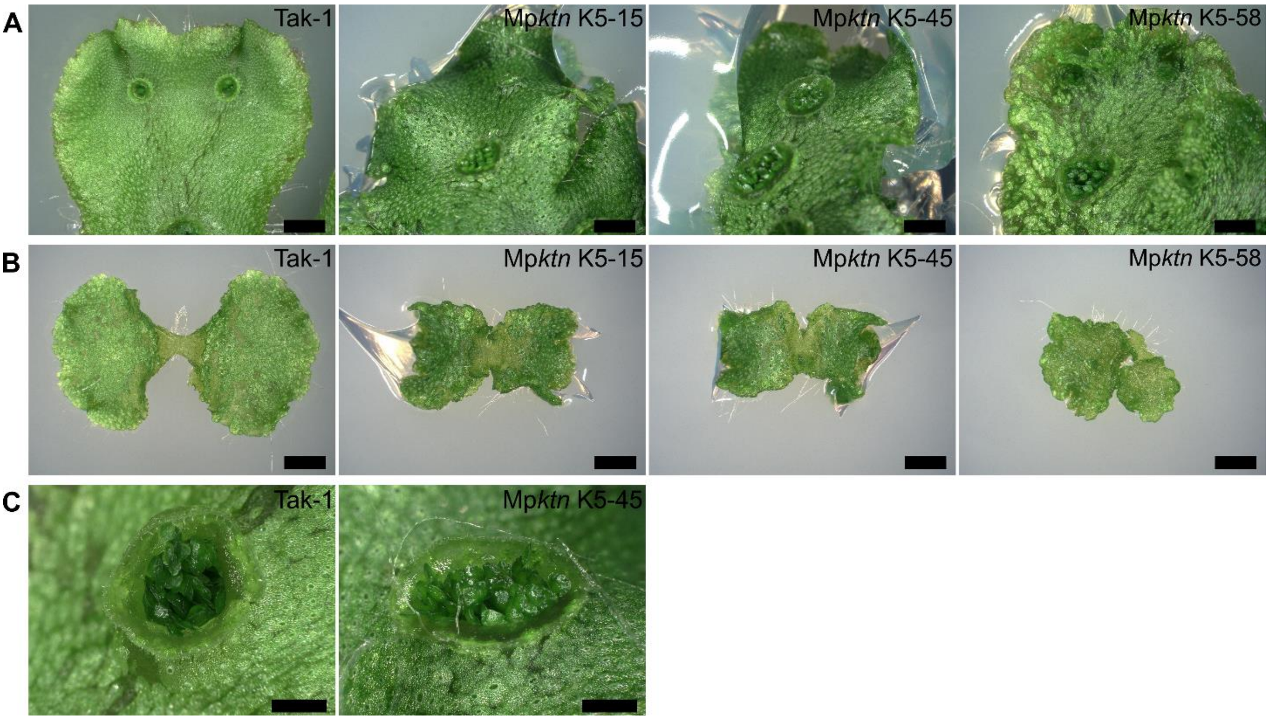

Fig. S2. Mutations in the encoded VSP4 domain of Mp*KTN* result in a defective *M. polymorpha* development similar to mutations in the encoded AAA domain of Mp*KTN*. Dorsal thallus (A), 10-day-old gemmalings (B) and gemma cups on dorsal thallus (C) from wild type (Tak-1) and Mp*ktn* lines with mutations in the encoded Vsp4c domain. Scale bars: 2 mm.

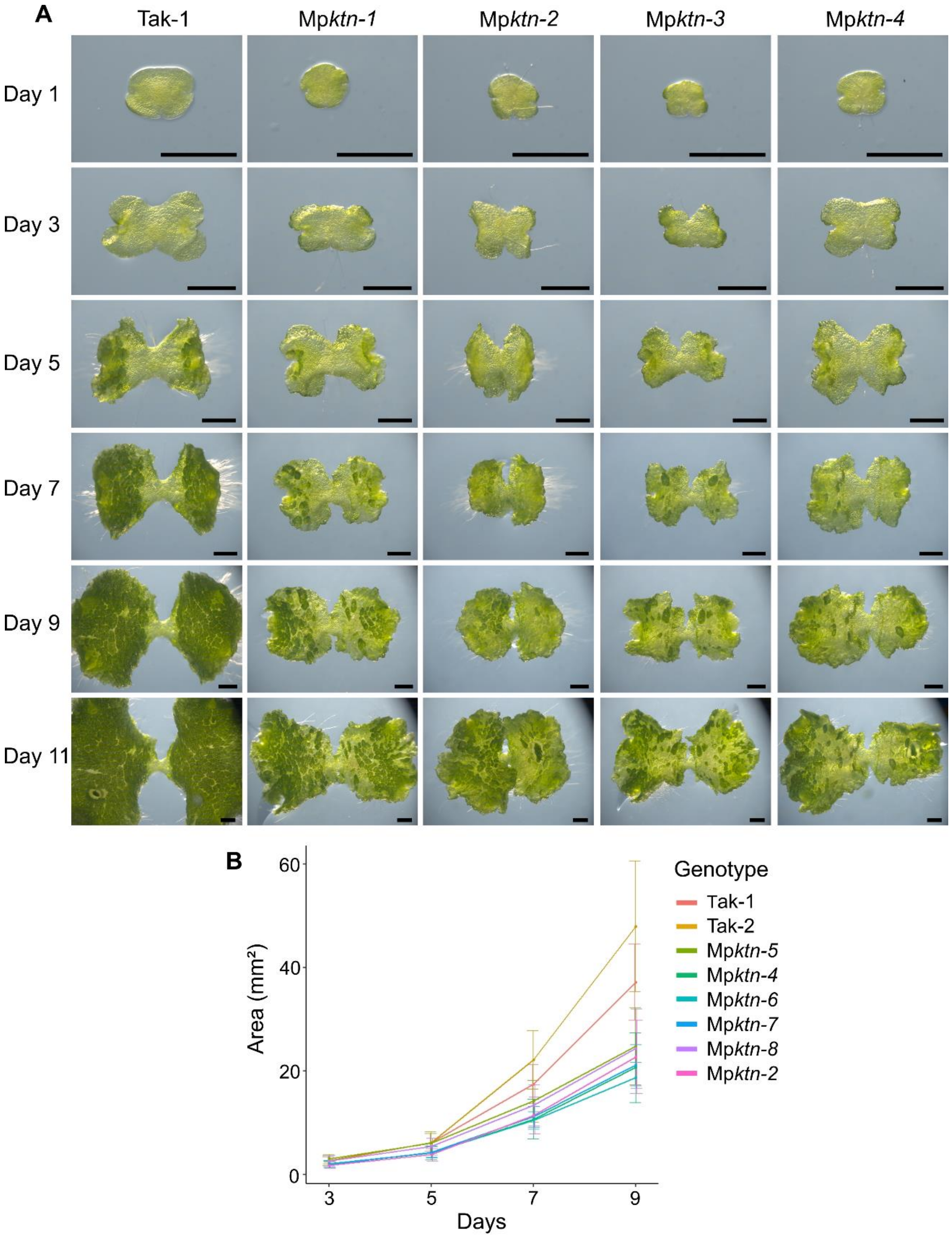

Fig. S3. Development of Mp*ktn* gemmalings is defective and slower than wild type.
(A) Development of gemmalings from wild type (Tak-1) and four Mp*ktn* lines over 11 days. Scale bars: 1 mm. (B) Plot of gemmaling tissue area over 9 days growth for wild type (Tak-1, Tak-2) and six Mp*ktn* lines with mutations in the encoded AAA ATPase domain. Tissue area was calculated from chlorophyll autofluorescence. Presented is the mean area (mm²) with standard error bars at each timepoint. Each genotype, *n*=9 plants.

Development • Supplementary information

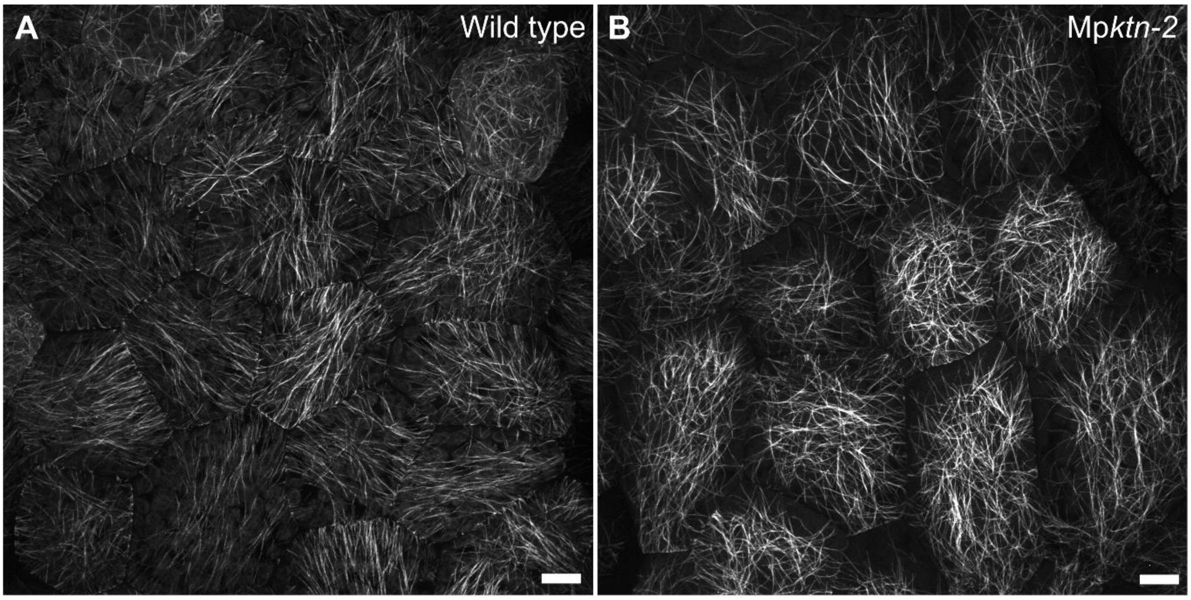

C

	Genotype	Mean	SD	CV	T-test (Welch's, 2-tail)	F-test (2-tail)
**Parallelness (NormAvgRad)**	Wild type	0.338	0.148	0.437	4.33E-18 ***	0.0001 ***
	Mp*ktn-1*	0.172	0.102	0.590		
**Bundling (skewness)**	Wild type	1.389	0.303	0.218	0.0121 *	0.0032 ***
	Mp*ktn-1*	1.297	0.227	0.175		
**Density (npix/µm²)**	Wild type	7.700	1.612	0.209	2.18E-11 ***	0.1350
	Mp*ktn-1*	9.384	1.864	0.199		
**Cell area (µm²)**	Wild type	895.851	286.589	0.320	0.8367	0.7932
	Mp*ktn-1*	887.883	279.375	0.315		
**Cell circularity**	Wild type	0.828	0.040	0.049	0.0658	5.31E-05 ***
	Mp*ktn-1*	0.815	0.060	0.073		

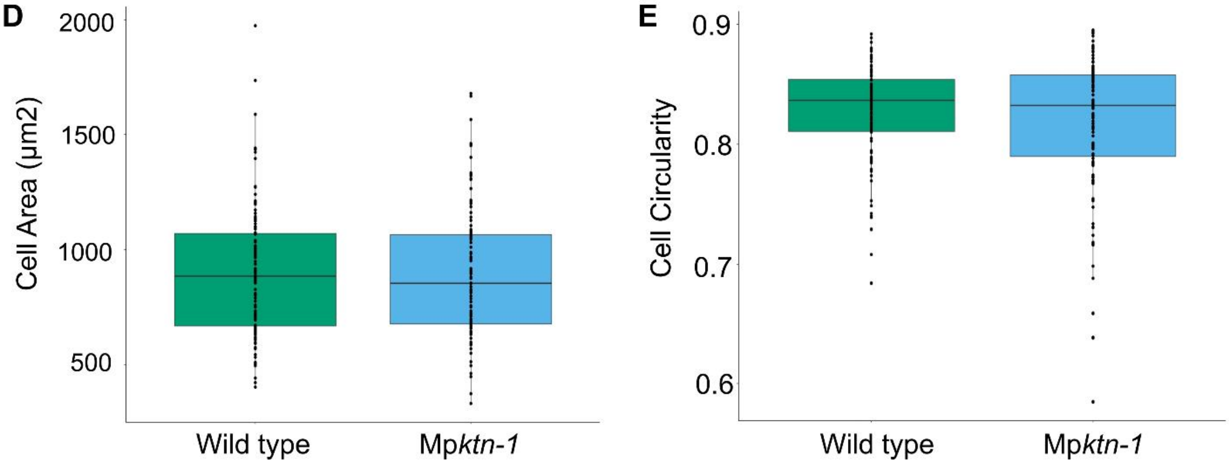

Fig. S4. Organisation of cortical arrays in wild type and Mp*ktn-1* gemmalings.

(A, B) Cortical microtubules in the central epidermal cells of wild type (A) and Mp*ktn-2* (B) 2-day-old gemmalings. Presented are *z*-projections. Scale bars: 10 µm. (C) Quantification of the parallelness, bundling and density of cortical microtubules in wild type and Mp*ktn-1* epidermal cells. The area and circularity of the cells was also quantified Wild type, *n*=108 cells; Mp*ktn-1, n*=107 cells. Cells from 10 gemmalings per genotype. Presented is the mean, standard deviation (SD) and co-efficient of variation (CV). Data was analysed using Welches 2-tail T-test and 2-tail F-test and significant differences between wild type and Mp*ktn-1* are indicated by * (P ≤ 0.05) and *** (P ≤ 0.001). (D, E) Boxplots of the mean area (D) and circularity (E) of wild type and Mp*ktn-1* epidermal cells as quantified in (C). Wild type, *n*=108 cells; Mp*ktn-1*, *n*=107 cells. Cells from 10 gemmalings per genotype.

Development • Supplementary information

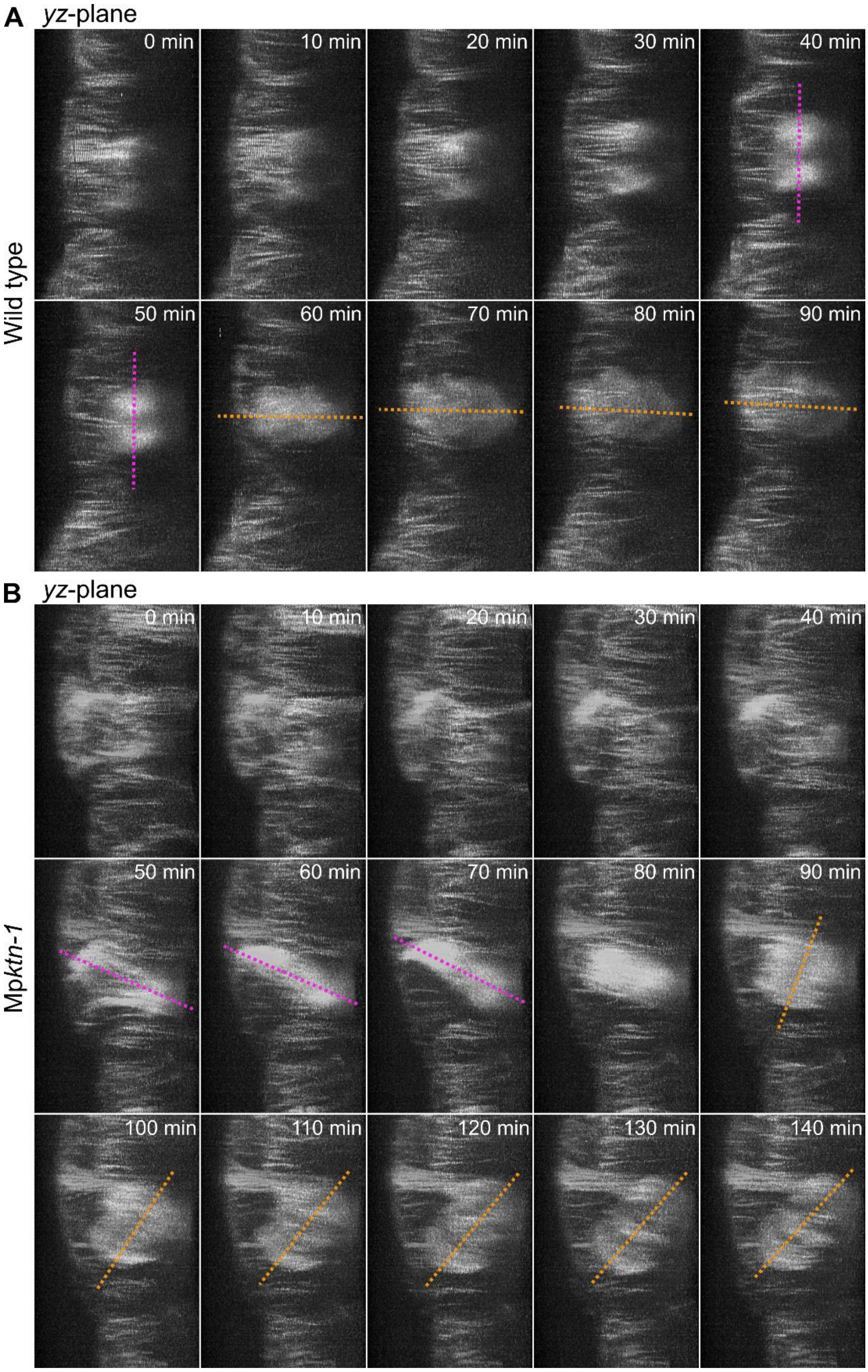
A
yz-plane
Wild type
0 min
10 min
20 min
30 min
40 min
50 min
60 min
70 min
80 min
90 min
B
yz-plane
Mpktn-1
0 min
10 min
20 min
30 min
40 min
50 min
60 min
70 min
80 min
90 min
100 min
110 min
120 min
130 min
140 min

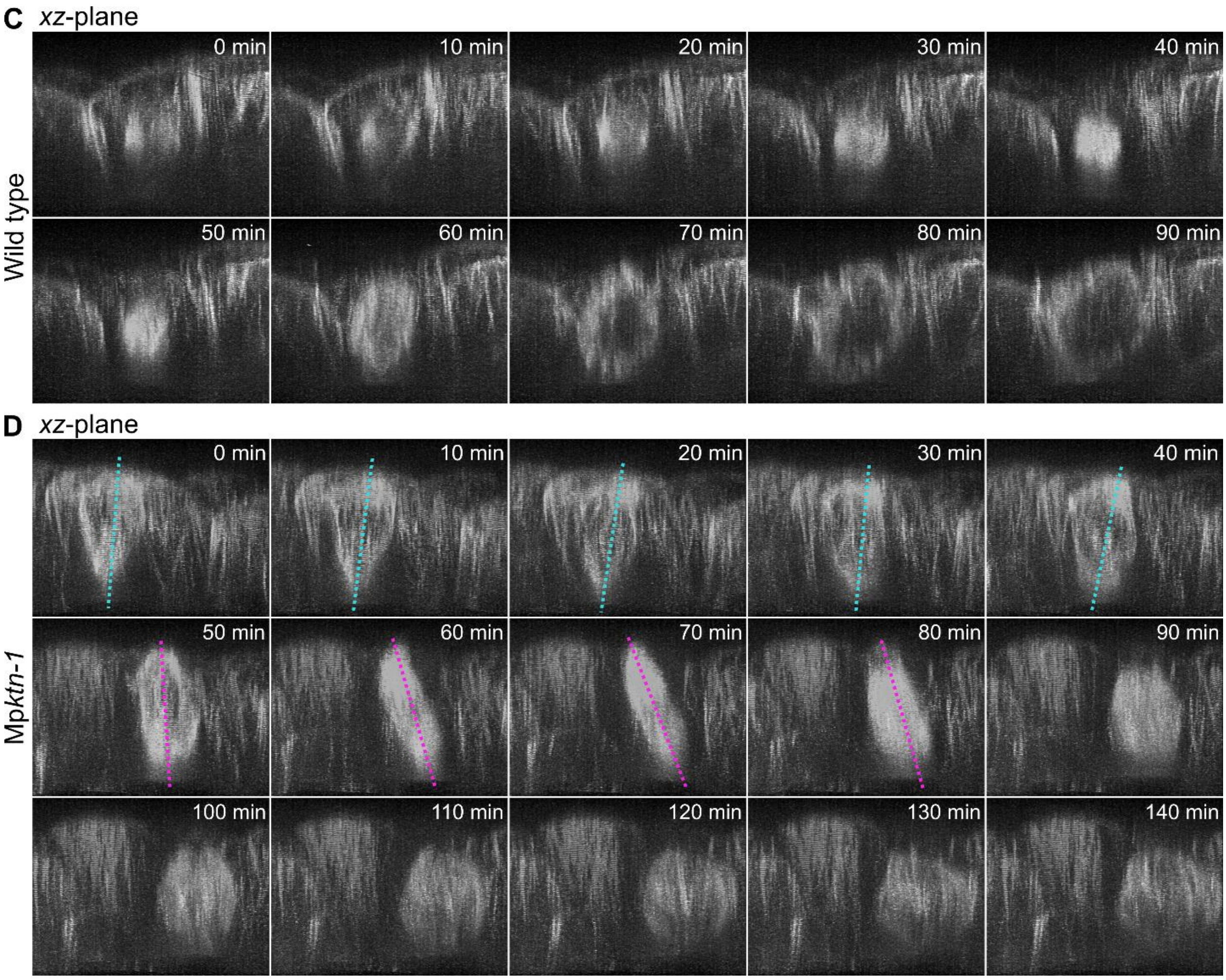

Fig. S5. Views from the *yz*- and *xz*-planes of dividing wild type and Mp*ktn-1* cells show the tilting of polar organisers, mitotic spindle and phragmoplast relative to the dorsal cell surface in Mp*ktn-1*. Timelapses of microtubule organisations in dividing wild type (A, C) and Mp*ktn-1* (B, D) cells (from Fig. 4A, B) viewed from the *yz*-plane (A, B) and *xz*-plane (C, D). Presented are deconvolved *z*-projections viewed from the *yz*- and *xz*-planes. Dotted cyan lines indicate the polar organiser axis, dotted magenta lines indicate the mitotic spindle axis and dotted orange lines indicate phragmoplast plane.

Development • Supplementary information

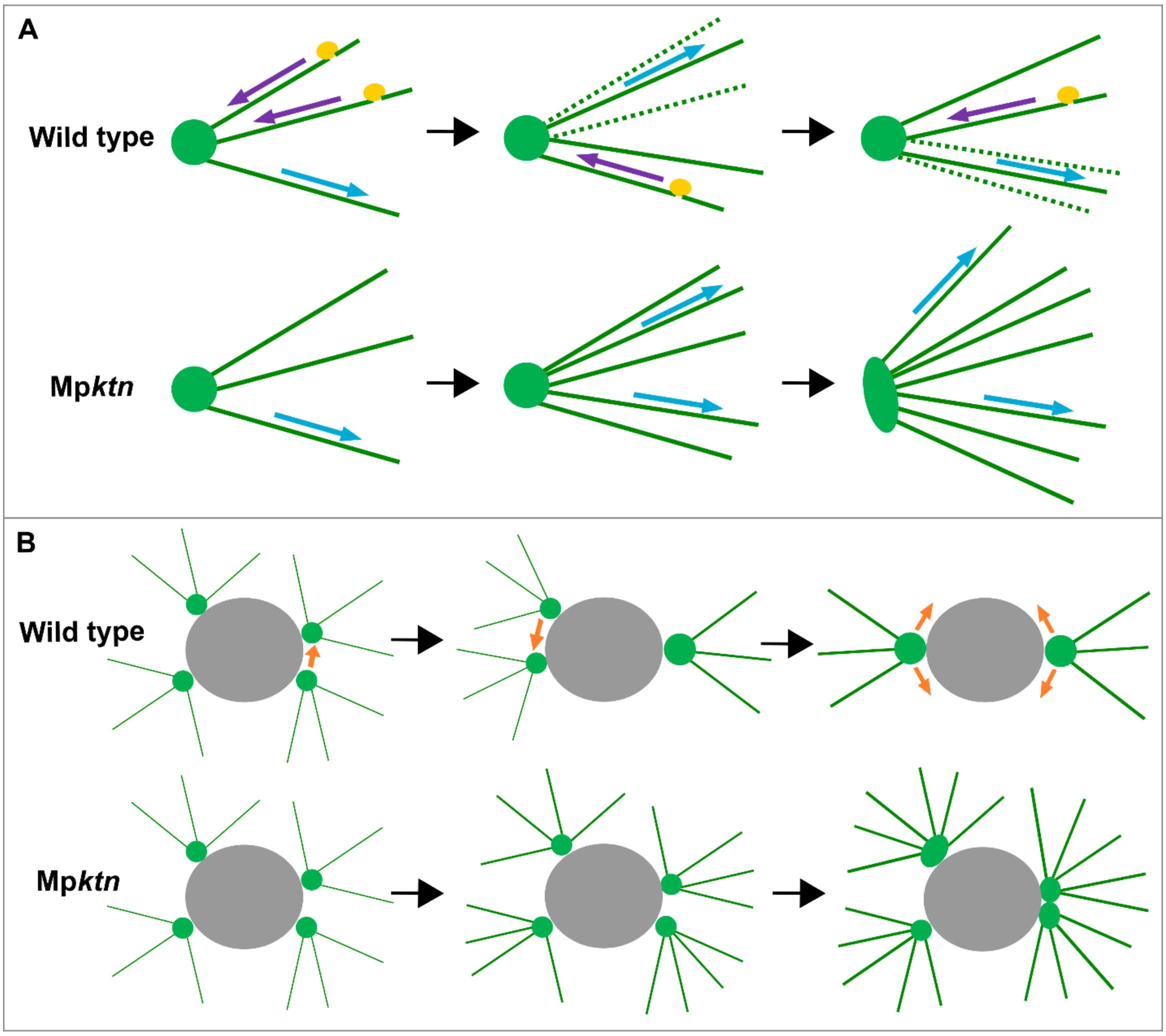

Fig. S6. Hypotheses for the role of KTN in astral microtubule dynamics and the formation of polar organisers in *Marchantia polymorpha*. (A) Hypothesis for the dynamics of astral microtubules in wild type and Mp*ktn*. Astral microtubules continuously polymerise from polar organisers. In wild type, KTN cuts some astral microtubules resulting in their depolymerisation. Astral array density remains stable over time. In Mp*ktn*, no astral arrays are severed and depolymerised. Astral array density increases over time and the polar organiser expands to accommodate. Green lines represent microtubules, green circles represent polar organisers and yellow circles represent KTN proteins. Blue arrows indicate microtubule polymerisation and purple arrows indicate microtubule depolymerisation after KTN severing. (B) Hypothesis for the formation of polar organisers in wild type and Mp*ktn*. Multiple microtubule nucleation sites (foci) form around the nucleus in preprophase. In wild type, these foci can move and fuse to form two polar organisers. In Mp*ktn*, these foci cannot move and fuse due to their stable astral arrays. Each foci becomes a polar organiser, leading to a multipolar structure. Green lines represent microtubules, green circles represent microtubule foci and polar organisers, and grey circles represent the nucleus. Orange arrows indicate the movement of microtubule foci and polar organisers.

Development • Supplementary information

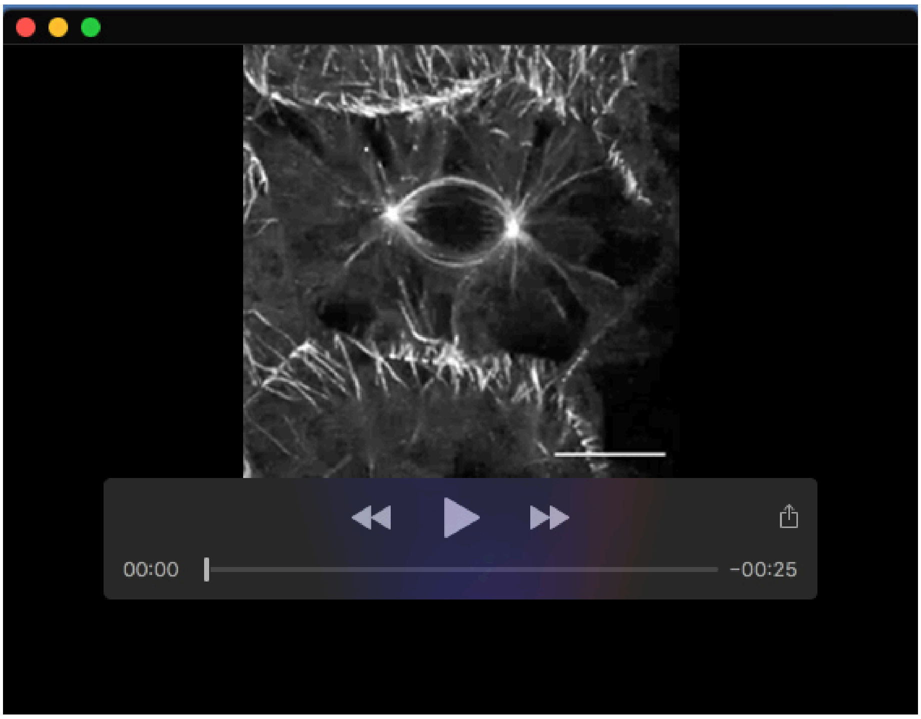

Movie 1. Dynamics of wild type polar organisers and astral arrays

Movie of polar organisers, astral microtubules, and perinuclear arrays in a wild type epidermal cell from a 2-day-old gemmaling. Presented are deconvolved Z-projections of slices from within the cell centre, taken at 8.13s time intervals. The two polar organisers are highly mobile, and the astral microtubules polymerise rapidly over the time course.

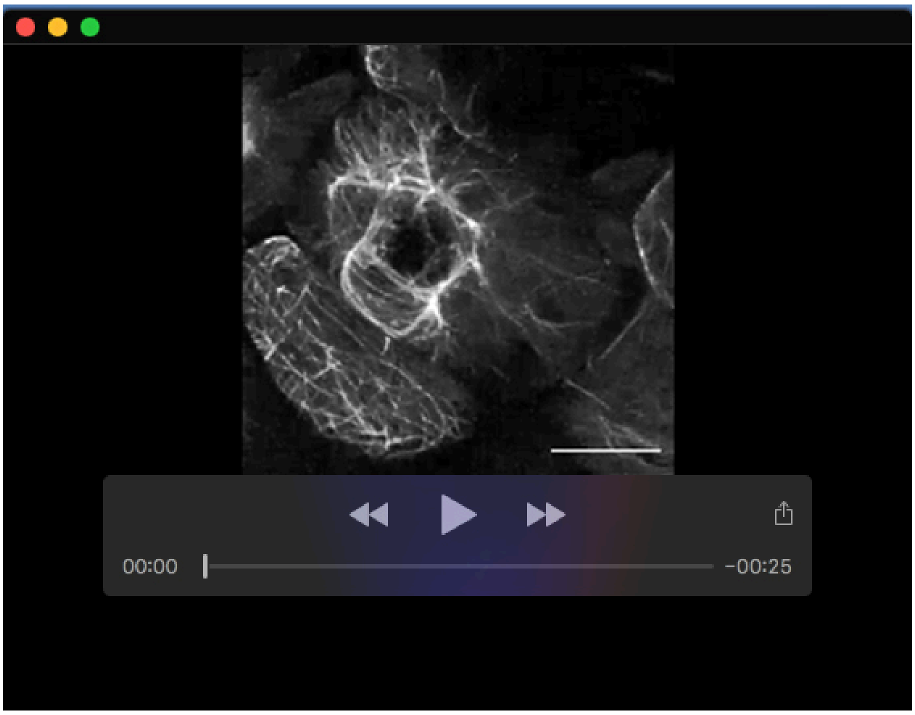

Movie 2. Dynamics of Mp*ktn-1* polar organisers and astral arrays

Movie of polar organisers, astral microtubules, and perinuclear arrays in a Mp*ktn-1* epidermal cell from a 2-day-old gemmaling. Presented are deconvolved Z-projections of slices from within the cell centre, taken at 8.13s time intervals. The polar organisers and astral microtubules appear stable over the time course.

Development • Supplementary information
