# Supplementary material for: KATANIN-mediated microtubule severing is required for MTOC organisation and function in Marchantia polymorpha
Source: Development. 2024 May 1;151(20):dev202672. doi: 10.1242/dev.202672 (PMC11112166; doi:10.1242/dev.202672)
Supplement: Supplementary file 1 [file develop-151-202672_review_history.pdf]

KATANIN-mediated microtubule severing is required for MTOC organisation and function in *Marchantia polymorpha*

Sarah T. Attrill and Liam Dolan
DOI: 10.1242/dev.202672

Editor: Dominique Bergmann

Review timeline
Original submission: 4 January 2024
Editorial decision: 8 February 2024
First revision received: 18 March 2024
Accepted: 20 March 2024

Original submission

First decision letter

MS ID#: DEVELOP/2024/202672

MS TITLE: KATANIN-mediated microtubule severing is required for MTOC formation and function in Marchantia polymorpha

AUTHORS: Sarah Thurza Attrill and Liam Dolan

I have now received all the referees reports on the above manuscript, and have reached a decision. The referees' comments are appended below, or you can access them online: please go to BenchPressand click on the 'Manuscripts with Decisions' queue in the Author Area.

The overall evaluation is positive and we would like to publish a revised manuscript in Development, provided that the referees' comments can be satisfactorily addressed. My editorial view of the reviewers assessment is that no new experimental data are needed (though if you have data to address some of the questions, you can certainly add it), but some data analysis and writing should be revised.

Please attend to all of the reviewers' comments in your revised manuscript and detail them in your point-by-point response. If you do not agree with any of their criticisms or suggestions explain clearly why this is so. If it would be helpful, you are welcome to contact us to discuss your revision in greater detail. Please send us a point-by-point response indicating your plans for addressing the referees' comments, and we will look over this and provide further guidance.

Reviewer 1

Advance summary and potential significance to field

KATANIN(KTN) is a well-studied microtubule-serving AAA protein affecting microtubule organization and dynamics in wide variety of organisms including animals and plants. KTN is a heterodimer that consists of a 60 kDa ATPase (p60 subunit A1) and an 80 kDa accessory protein (p80 subunit B1). In angiosperm, KTN has been shown to be involved in cortical microtubule organization during establishment of cell growth directionality. KTN is also suggested to function in the mitosis and the preprophase band (PPB)-determined cell division plane orientation. Microtuble organizing centers

© 2024. Published by The Company of Biologists under the terms of the Creative Commons Attribution License (https://creativecommons.org/licenses/by/4.0/).

(MTOCs), which includes centrioles, are not formed in the somatic cells of vascular plants, but observed in bryophytes. In a bryophyte model, Marchantia polymorpha, two MTOCs form at opposite sides of the prophase nucleus. In this study, the authors hypothesized that KTN-mediated microtubule serving plays pivotal role in the formation of MTOC, polar organizers, in bryophytes, and studied the KTN function in M. polymorpha. The authors generated loss of function mutants of Marchantia homolog of KTN, MpKTN (Mp4g20260), and underwent a series of developmental and cell biological characterizations also using microtubule reporter. This study has led a certain conclusion that KTN-mediated microtubule dynamics are required for the de novo formation of MTOC which is critical for cell division plane orientation in M. polymorpha. To my knowledge, this is the novel function of KTN in land plants. This study is also important for our better understanding of the mechanisms how the orientation of cell division plane is determined in plants. The manuscript is generally clear and well written.

Comments for the author

To improve the manuscript, I suggest the authors to revise the manuscript in response to the following points.

<General points>
MTOC formation is also involved in the motile spermatozoids in M. polymorpha. I would like to see the MTOC formation during the sperm development, and also the sperm functionality in the wild type and Mpktn mutants.

Introduction is a bit too concise to learn about the background of KTN for general readers. The authors should describe more about biochemical backgrounds of KTN, and also some more information about KTN function in animals especially the role in centriole organization.

<Minor points>
Page 8, line 2-14 M. polymorpha encodes only a single gene for KTN p80. How is the expression pattern of MpKTN(p60) in various tissues, developmental stages and environmental conditions? The authors should provide some data/information about the expression pattern of MpKTN.
It would be also nicer to provide information about Marchantia homologus of KTN p80 subunit.

Page 10, line 9-18 or Page 7, line 7-10 The authors nicely quantified the microtubule organization in Marchantia cells, but does not describe any information about the principle of quantification. Please add some description about the quantification of microtubule organization; e.g. skewness of the fluorescence intensity distribution is considered/used as a good indicator of microtubule bundling as showed in a certain reference(s).

Page 15, line 14-
I cannot understand the sentence “The polar organiser axis was parallel to the cell surface and perpendicular to the PPB”. Theoretically as a cube, there are six surfaces in a cell. Please define the surface to which the polar organizer is parallel to.

<u>Reviewer 2</u>

Advance summary and potential significance to field

The manuscript by Attrill and Dolan for the first time report the role of KATANIN in Marchantia polymorpha, with a focus on its importance during formation of microtubule organizing centers and subsequent cell division machinery. Overall the manuscript is well written and provides novel insights in the development of Marchantia. I only have minor suggestions.

Comments for the author

1. The title indicates the formation of MTOC, however, I feel that it is rather the organization of the poles that are impacted based on the evidence provided here. Currently, there is no data in the manuscript that points towards formation of MTOCs and the hypothesis is speculative as indicated by the authors in the final part of the manuscript.

© 2024. Published by The Company of Biologists under the terms of the Creative Commons Attribution License (https://creativecommons.org/licenses/by/4.0/).

2. The authors indicate that KATANIN is important for cell-specific microtubule organization, not sure, how the authors can conclude this as no information on the cell type investigated is provided. I agree that there is less variability in microtubule organization but this is not sufficient to claim cell-type specificity.
3. Related to the above point the authors measure variability using standard deviation; however, the use of coefficient of variation is better for such analysis with proper statistical comparison.
4. It should be noted that the PPB fine tunes the positioning of the new cell wall (Schaefer et al., Science 2007) rather than predicting the future cell division plane as indicated in the text.
5. Figure 3F the cell size/shape is drastically different between the wildtype and the mutant, has this been taken into account? Please provide data from comparable cell size/shapes.
6. The potential off centering of the mitotic spindle could be due to differences in cell size/shapes. It is well know that cell shape related cues act to position the cell division apparatus and the effects seen here could be due to more isotropic cells shapes observed in the mutant. This should at least be discussed in the manuscript. This is also true to Fig 4a and b.
7. The authors indicate that the polar organizer's axis is not parallel to the cell surface. Cell surface is not the correct term; I think it is better to state that they are parallel to the longest axis of the cell. Further, in the mutant the polar organizer's axis parallel to one of the anticlinal walls so I would suggest authors to rephrase this.

First revision

Author response to reviewers' comments

MS ID#: DEVELOP/2024/202672
MS TITLE: KATANIN-mediated microtubule severing is required for MTOC organisation and function in Marchantia polymorpha

AUTHORS: Sarah Thurza Attrill and Liam Dolan

Response to Reviewers

Reviewer 1 Advance Summary and Potential Significance to Field...

KATANIN (KTN) is a well-studied microtubule-serving AAA protein affecting microtubule organization and dynamics in wide variety of organisms including animals and plants. KTN is a heterodimer that consists of a 60 kDa ATPase (p60 subunit A1) and an 80 kDa accessory protein (p80 subunit B1). In angiosperm, KTN has been shown to be involved in cortical microtubule organization during establishment of cell growth directionality. KTN is also suggested to function in the mitosis and the preprophase band (PPB)-determined cell division plane orientation. Microtuble organizing centers (MTOCs), which includes centrioles, are not formed in the somatic cells of vascular plants but observed in bryophytes. In a bryophyte model, Marchantia polymorpha, two MTOCs form at opposite sides of the prophase nucleus. In this study, the authors hypothesized that KTN-mediated microtubule serving plays pivotal role in the formation of MTOC, polar organizers, in bryophytes, and studied the KTN function in M. polymorpha. The authors generated loss of function mutants of Marchantia homolog of KTN, MpKTN (Mp4g20260), and underwent a series of developmental and cell biological characterizations also using microtubule reporter. This study has led a certain conclusion that KTN-mediated microtubule dynamics are required for the de novo formation of MTOC which is critical for cell division plane orientation in M. polymorpha. To my knowledge, this is the novel function of KTN in land plants. This study is also important for our better understanding of the mechanisms how the orientation of cell division plane is determined in plants. The manuscript is generally clear and well written.

Reviewer 1 Comments for the Author...

© 2024. Published by The Company of Biologists under the terms of the Creative Commons Attribution License (https://creativecommons.org/licenses/by/4.0/).

To improve the manuscript, I suggest the authors to revise the manuscript in response to the following points.

COMMENT 1:
MTOC formation is also involved in the motile spermatozoids in M. polymorpha. I would like to see the MTOC formation during the sperm development, and also the sperm functionality in the wild type and Mpktn mutants.

RESPONSE 1:
This is an interesting question, but we felt it was beyond the scope of the current paper.

COMMENT 2:
Introduction is a bit too concise to learn about the background of KTN for general readers. The authors should describe more about biochemical backgrounds of KTN, and some more information about KTN function in animals especially the role in centriole organization.

RESPONSE 2:
We have included more information in the Introduction to address this comment.

COMMENT 3:
Page 8, line 2-14. M. polymorpha encodes only a single gene for KTN p80. How is the expression pattern of MpKTN(p60) in various tissues, developmental stages and environmental conditions? The authors should provide some data/information about the expression pattern of MpKTN.
It would be also nicer to provide information about Marchantia homologues of KTN p80 subunit.

RESPONSE 3:
MpKTN p60 (Mp4g20260) and MpKTN p80 (Mp3g07080) are both expressed across all tissues and developmental stages, as seen in the Marchantia expression atlas (Marpolbase, marchantia.info/mbex). As we have focused on the catalytic p60 subunit in this paper, we have not characterized the function of the p80 subunit.

COMMENT 4:
Page 10, line 9-18 or Page 7, line 7-10. The authors nicely quantified the microtubule organization in Marchantia cells but does not describe any information about the principle of quantification. Please add some description about the quantification of microtubule organization; e.g. skewness of the fluorescence intensity distribution is considered/used as a good indicator of microtubule bundling as showed in a certain reference(s).

RESPONSE 4:
We have added more detail into the Material and methods about each parameter, and a few sentences to the Results to concisely describe these principles.

COMMENT 5:
Page 15, line 14. I cannot understand the sentence “The polar organiser axis was parallel to the cell surface and perpendicular to the PPB”. Theoretically as a cube, there are six surfaces in a cell. Please define the surface to which the polar organizer is parallel to.

RESPONSE 5:
Thank you for pointing out the lack of clarity in this sentence. We have rewritten it and referred to the ‘dorsal cell surface’.

© 2024. Published by The Company of Biologists under the terms of the Creative Commons Attribution License (https://creativecommons.org/licenses/by/4.0/).

Reviewer 2 Advance Summary and Potential Significance to Field...

The manuscript by Attrill and Dolan for the first time report the role of KATANIN in Marchantia polymorpha, with a focus on its importance during formation of microtubule organizing centers and subsequent cell division machinery. Overall, the manuscript is well written and provides novel insights in the development of Marchantia. I only have minor suggestions.

Reviewer 2 Comments for the Author...

COMMENT 1:
The title indicates the formation of MTOC, however, I feel that it is rather the organization of the poles that are impacted based on the evidence provided here. Currently, there is no data in the manuscript that points towards formation of MTOCs and the hypothesis is speculative as indicated by the authors in the final part of the manuscript.

RESPONSE 1:
We changed the title and wording throughout the text from 'the formation of MTOCs' to 'the organisation of MTOCs' in view of this comment.

COMMENT 2:
The authors indicate that KATANIN is important for cell-specific microtubule organization, not sure, how the authors can conclude this as no information on the cell type investigated is provided. I agree that there is less variability in microtubule organization but this is not sufficient to claim cell-type specificity.

RESPONSE 2:
Thank you for spotting this error. Of course, it is not cell type specific. We have modified the text.

COMMENT 3:
Related to the above point the authors measure variability using standard deviation; however, the use of coefficient of variation is better for such analysis with proper statistical comparison.

RESPONSE 3:
We have added coefficient of variation to the table in SFig.4.

COMMENT 4:
It should be noted that the PPB fine tunes the positioning of the new cell wall (Schaefer et al., Science 2007) rather than predicting the future cell division plane as indicated in the text.

RESPONSE 4:
We have modified our text to highlight this point and added this reference.

The modified text now reads: "The preprophase band (PPB) is a parallel microtubule array that forms in the cell cortex during prophase – after polar organiser formation – and fine tunes the positioning of the division plane (Brown and Lemmon, 1990; Rasmussen et al., 2013; Schaefer et al., 2017)."

COMMENT 4:
Figure 3F the cell size/shape is drastically different between the wildtype and the mutant, has this been taken into account? Please provide data from comparable cell size/shapes.

RESPONSE 4:

© 2024. Published by The Company of Biologists under the terms of the Creative Commons Attribution License (https://creativecommons.org/licenses/by/4.0/).

We have added data on the size and shape of wild type and mutant cells to SFig4. The mean cell size and shape are not significantly different between mutant and wild type, but cell shape is more variable in the mutant. Therefore, the size and shape of epidermal cells does not account for the differences in cortical microtubule organisation between mutant and wild type.

COMMENT 5:
The potential off centering of the mitotic spindle could be due to differences in cell size/shapes. It is well known that cell shape related cues act to position the cell division apparatus and the effects seen here could be due to more isotropic cells shapes observed in the mutant. This should at least be discussed in the manuscript. This is also true to Fig 4a and b.

RESPONSE 5:
This could be part of the explanation, but the polar organisers are often located very close to one side of the cell in the mutant which cannot be explained by the cell geometry – it is as though the polar organisers ignore cell geometry in the Mp*ktn* mutant background. We have added an explanation to the text and exchanged image G to clearly demonstrate the spindle mispositioning often seen in the mutant.

COMMENT 6:
The authors indicate that the polar organizer's axis is not parallel to the cell surface. Cell surface is not the correct term; I think it is better to state that they are parallel to the longest axis of the cell. Further, in the mutant the polar organizer's axis parallel to one of the anticlinal walls so I would suggest authors to rephrase this.

RESPONSE 6:
Thanks for pointing this out – we agree that our statement was incorrect. We have changed the text to specify the 'dorsal cell surface' and related the polar organiser axis to this surface.

Second decision letter

MS ID#: DEVELOP/2024/202672

MS TITLE: KATANIN-mediated microtubule severing is required for MTOC organisation and function in Marchantia polymorpha

AUTHORS: Sarah Thurza Attrill and Liam Dolan

I am happy to tell you that your manuscript has been accepted for publication in Development, pending our standard ethics checks.

© 2024. Published by The Company of Biologists under the terms of the Creative Commons Attribution License (https://creativecommons.org/licenses/by/4.0/).
